# Orforglipron: A Comprehensive Review of an Oral Small-Molecule GLP-1 Receptor Agonist for Obesity and Type 2 Diabetes

**DOI:** 10.3390/ijms27031409

**Published:** 2026-01-30

**Authors:** Urna Kansakar, Stanislovas S. Jankauskas, Shivangi Pande, Pasquale Mone, Fahimeh Varzideh, Gaetano Santulli

**Affiliations:** 1 School of Medicine, City University of New York, Manhattan, NY 10031, USA; 2 Casa di Cura “Clinica Montevergine”, 83013 Mercogliano, Italy

**Keywords:** cardiology, diabetes mellitus, GLP-1, obesity, orforglipron, metabolism

## Abstract

Orforglipron (LY3502970) is a novel, orally available, nonpeptide glucagon-like peptide-1 receptor agonist (GLP-1 RA) designed to replicate the efficacy of injectable GLP-1 RAs for glycemic control and weight reduction while improving convenience and adherence. Preclinical studies have demonstrated potent receptor engagement, favorable pharmacokinetics, and central nervous system activity. Phase 1–3 clinical trials have shown significant reductions in glycated hemoglobin (HbA1c), fasting and postprandial glucose, body weight, and cardiovascular risk biomarkers, with an acceptable safety profile. This comprehensive review integrates pharmacological, clinical, and mechanistic evidence, critically evaluates the data, identifies knowledge gaps, and outlines future directions for orforglipron in the treatment of type 2 diabetes and obesity.

## 1. Introduction

Type 2 diabetes mellitus (T2D) and obesity are interrelated chronic conditions characterized by insulin resistance, β-cell dysfunction, hyperglycemia, and dysregulated energy balance. These disorders are associated with increased morbidity and mortality, largely driven by cardiovascular disease, renal impairment, and metabolic complications. Despite multiple therapeutic options, adherence and long-term effectiveness remain suboptimal, in part due to the need for injectable medications, polypharmacy, and gastrointestinal side effects [1].

The glucagon-like peptide-1 receptor (GLP-1R) is a G protein-coupled receptor belonging to the class B (secretin family) GPCRs [2,3,4,5,6,7,8]. It primarily couples to Gs proteins, leading to activation of adenylyl cyclase, increased intracellular cAMP, and downstream signaling through PKA and Epac [9,10,11,12]. In a cell type- and context-dependent manner, GLP-1R can also engage β-arrestin-mediated pathways and limited coupling to other G proteins, contributing to biased signaling. Through these mechanisms, GLP-1R activation enhances glucose-stimulated insulin secretion; suppresses glucagon release; slows gastric emptying; promotes satiety; and exerts cardiometabolic effects relevant to obesity, heart failure, and chronic kidney disease [13,14,15,16].

Glucagon-like peptide-1 receptor agonists (GLP-1 RAs) have emerged as a cornerstone in the management of T2D and obesity, providing glucose-dependent insulin secretion, glucagon suppression, delayed gastric emptying, appetite reduction, and cardiometabolic benefits [17,18,19,20,21,22,23]. Oral formulations are highly desirable to improve patient acceptance and broaden therapeutic reach [24].

Orforglipron represents a first-in-class, nonpeptide small-molecule GLP-1 RA designed for oral administration [25,26,27,28,29,30,31]. Its development addresses the unmet need for effective, patient-friendly GLP-1-based therapy. This comprehensive review summarizes current evidence on orforglipron, including pharmacological characteristics, glycemic and weight-loss efficacy, safety, cardiovascular effects, and potential clinical positioning. Special emphasis is placed on mechanistic insights, critical appraisal of clinical trials, and identification of gaps and future research directions.

## 2. Pharmacological Basis of Orforglipron

### 2.1. Molecular Structure and Mechanism

Orforglipron (LY3502970) is a synthetic, orally bioavailable, nonpeptide agonist of the GLP-1 receptor that was rationally designed to overcome the intrinsic limitations of peptide-based GLP-1 RAs, particularly poor oral bioavailability and rapid enzymatic degradation [28,29,32,33]. As a small molecule, orforglipron is chemically stable in the acidic gastric environment and is not a substrate for proteolytic enzymes such as dipeptidyl peptidase-4, enabling efficient absorption from the gastrointestinal tract after oral administration and eliminating the need for absorption enhancers or complex formulation strategies that are required for oral peptide GLP-1 therapies [33,34,35]. This pharmacochemical property is central to its once-daily oral dosing and represents a substantial advance in the development of patient-friendly incretin-based treatments [36].

**Figure 1:**
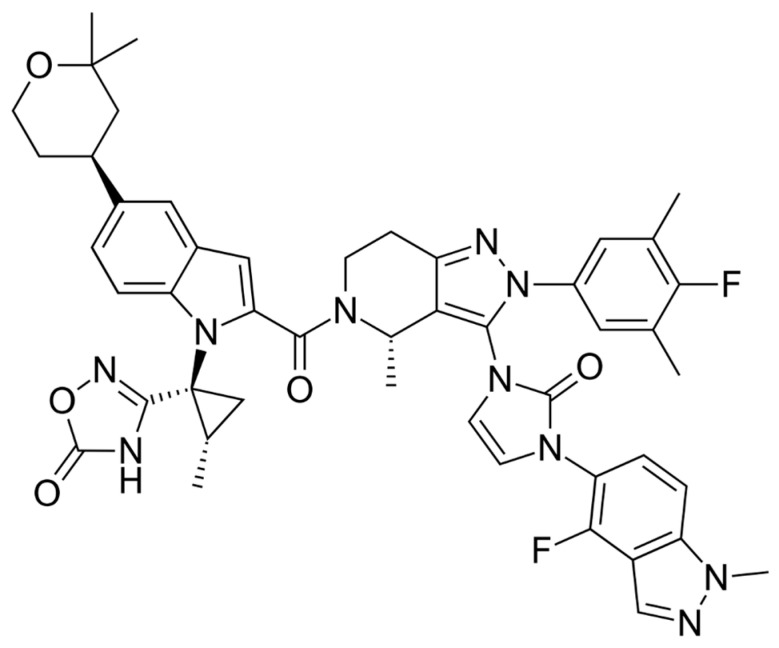
Chemical structure of orforglipron.

At the structural level, orforglipron is built around a rigid, polycyclic heteroaromatic scaffold that confers high conformational stability and resistance to enzymatic degradation in the gastrointestinal tract [30,32,33,36,37,38,39]. The molecule incorporates multiple aromatic and heteroaromatic rings, including nitrogen-containing moieties, which enable precise engagement with a hydrophobic pocket within the transmembrane domain of the GLP-1 receptor rather than the extracellular peptide-binding region; this architecture is critical for its allosteric binding mode, allowing receptor activation without mimicking the native peptide sequence (Figure 1).

**Figure 2:**
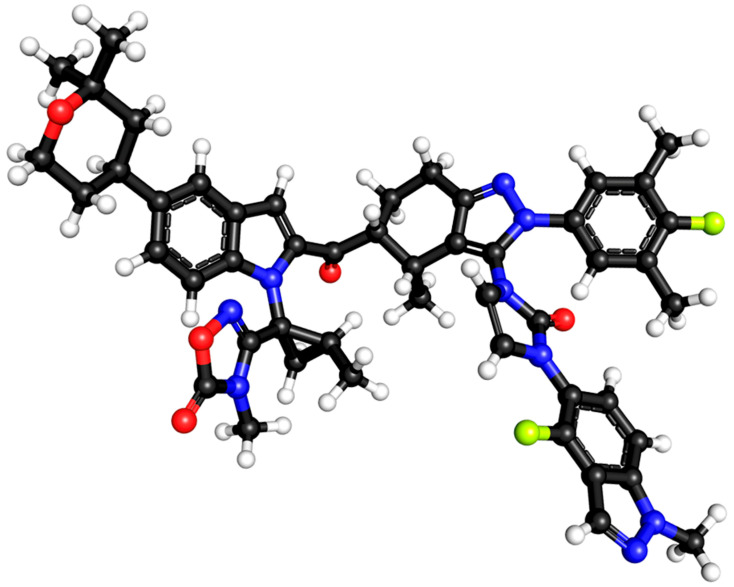
Chemical (ball-and-stick) structure of orforglipron, dominated by interconnected aromatic and heteroaromatic rings forming a dense three-dimensional core. Black spheres represent carbon atoms; red spheres are oxygen atoms, green spheres represent fluorine (F) atoms, and white spheres are hydrogen atoms; nitrogen atoms (blue) are embedded within heterocycles and serve as key polar interaction points. Short aliphatic linkers connect aromatic domains, creating a balanced geometry that combines lipophilic planar regions with polar protrusions. The absence of long flexible chains or peptide-like backbones results in a relatively globular, non-extended shape, well suited for insertion into the transmembrane allosteric pocket of the GLP-1 Receptor.

The compound contains strategically positioned polar functional groups that balance aqueous solubility with membrane permeability, a key requirement for efficient oral absorption. These polar elements participate in hydrogen bonding and electrostatic interactions within the receptor binding pocket, while surrounding lipophilic regions promote favorable pharmacokinetics and sustained GLP-1 receptor engagement (Figure 2).

The overall structure of orforglipron reflects careful optimization to achieve sufficient oral exposure, receptor potency, and metabolic stability without reliance on large peptide chains or fatty-acid conjugation strategies used in injectable GLP-1 RAs. Importantly, orforglipron lacks peptide bonds entirely, which eliminates susceptibility to proteases such as DPP-4 and neutral endopeptidases. This chemical feature not only supports oral delivery but also contributes to predictable pharmacokinetics and low interindividual variability.

Mechanistically, orforglipron differs fundamentally from peptide GLP-1 RAs in its mode of receptor engagement. Whereas endogenous GLP-1 and peptide agonists bind primarily to the orthosteric binding pocket of the GLP-1 receptor, orforglipron acts as a nonpeptide allosteric agonist [40]. High-resolution structural, mutagenesis, and signaling studies demonstrate that orforglipron binds within a transmembrane pocket distinct from the peptide-binding domain, stabilizing an active receptor conformation that efficiently couples to Gs proteins and drives intracellular cAMP accumulation. Indeed, orforglipron binds within the transmembrane core of the receptor rather than the extended extracellular domain used by the native GLP-1 peptide. Structural and modeling data indicate that it occupies a pocket formed primarily by TM1, TM2, TM3, TM7, and ECL2, engaging key hydrophobic residues in TM3 and TM7 and stabilizing an active receptor conformation. This binding site partially overlaps with the orthosteric peptide pocket but extends into a distinct transmembrane sub-pocket, a feature thought to underlie the G_s_-biased signaling profile of orforglipron compared with other GLP-1R agonists [40]. This allosteric mode of activation recapitulates the canonical downstream signaling of GLP-1 receptor activation while offering greater chemical flexibility and oral bioavailability. Importantly, biased signaling analyses indicate that orforglipron preferentially activates pathways associated with metabolic efficacy rather than receptor internalization, which may contribute to sustained pharmacodynamic effects despite once-daily dosing.

Activation of the GLP-1 receptor by orforglipron translates into the full spectrum of physiological actions classically associated with incretin signaling. At the pancreatic level, cAMP-mediated signaling enhances glucose-dependent insulin secretion from β-cells while suppressing inappropriate glucagon release from α-cells, thereby reducing both fasting and postprandial glycemia. In the gastrointestinal tract, orforglipron slows gastric emptying, attenuating the rate of glucose absorption and blunting postprandial glucose excursions. In parallel, central nervous system penetration enables engagement of hypothalamic and brainstem circuits involved in appetite regulation and satiety, leading to reduced caloric intake and clinically meaningful weight loss. The glucose dependency of these effects is preserved, which explains the consistently low incidence of hypoglycemia observed across clinical trials in the absence of concomitant insulin or insulin secretagogues.

Preclinical pharmacology studies further demonstrate that orforglipron exhibits high selectivity for the GLP-1 receptor, with minimal off-target activity across a broad panel of G protein–coupled receptors, ion channels, and enzymes, thereby supporting a favorable safety profile. In vitro and in vivo potency is comparable to that of established peptide GLP-1 RAs, with robust stimulation of insulin secretion and appetite suppression in animal models of diabetes and obesity. Importantly, pharmacokinetic studies in preclinical species predicted a half-life compatible with once-daily oral dosing in humans, a prediction that has been confirmed in Phase 1 clinical trials assessing disposition and absolute bioavailability [41].

Collectively, these pharmacological characteristics position orforglipron as a first-in-class oral small-molecule GLP-1 RA that successfully bridges the gap between the potent metabolic efficacy of injectable incretin therapies and the convenience of oral administration. Its nonpeptide, allosteric mechanism of receptor activation not only enables oral delivery but also provides important proof of concept that small molecules can achieve full agonism of complex peptide hormone receptors. This represents a major advance in patient-centered therapy for T2D and obesity and establishes a platform for the future development of orally active incretin-based agents.

### 2.2. Pharmacokinetics and Pharmacodynamics

Pharmacokinetic studies in healthy volunteers and participants with T2D show that orforglipron has rapid absorption, dose-proportional exposure, and a half-life supportive of once-daily administration [41]. Peak plasma concentrations are typically achieved within 2–4 h, and oral bioavailability is approximately 30–40%. Food intake has minimal impact on systemic exposure, providing flexibility for administration in real-world settings [42]. The main pharmacokinetic studies are summarized in Table 1.

Pharmacodynamic effects are consistent with GLP-1 receptor activation. Early-phase insulin secretion is enhanced, glucagon is suppressed in a glucose-dependent manner, and postprandial glucose excursions are attenuated. Dose-dependent reductions in fasting glucose, HbA1c, and body weight have been observed, while hypoglycemia is rare except in patients receiving concomitant insulin secretagogues. Multiple ascending dose studies confirm predictable pharmacokinetics across a broad range of doses and populations, including Japanese participants [43].

Mechanistic substudies suggest that orforglipron engages central appetite pathways similarly to peptide GLP-1 RAs, contributing to reductions in caloric intake and body weight independent of glucose lowering [44]. The combination of pancreatic and central effects supports its dual role in glycemic control and weight management.

## 3. Glycemic Efficacy

### 3.1. Mechanistic Rationale

Orforglipron exerts glucose-lowering effects through multiple complementary mechanisms. By activating GLP-1 receptors on pancreatic β-cells, it enhances glucose-dependent insulin secretion, thereby improving both fasting and postprandial glycemia without causing hypoglycemia in isolation. Simultaneously, it suppresses glucagon secretion from α-cells, reducing hepatic glucose output. Central nervous system effects reduce appetite and caloric intake, indirectly contributing to improved glycemia through weight reduction [27]. This multifaceted mechanism mirrors that of injectable GLP-1 RAs but is achieved with the convenience of oral delivery.

### 3.2. Phase 2 Dose-Finding Studies

Phase 2 clinical trials evaluated dose–response relationships in participants with T2D inadequately controlled with diet, exercise, or metformin (Table 2).

Once-daily orforglipron produced significant, dose-dependent reductions in HbA1c, with higher doses achieving reductions exceeding 1.5 percentage points at 26 weeks [26]. Fasting and postprandial glucose levels decreased in parallel, and indices of β-cell function, including HOMA-B and insulinogenic index, improved significantly, demonstrating restoration of early-phase insulin secretion.

Safety assessments indicated that gastrointestinal adverse events, primarily nausea and diarrhea, were dose-dependent but generally mild and transient. These findings established the therapeutic window and informed dose selection for subsequent Phase 3 trials.

### 3.3. Phase 3 Evidence

The ACHIEVE-1 trial assessed orforglipron in early T2D inadequately controlled early T2D patients despite lifestyle interventions. Over 40 weeks, treatment resulted in substantial reductions in HbA1c (mean absolute reduction ~1.4–1.6%) without significant hypoglycemia [27]. Improvements were consistent across baseline HbA1c, BMI, sex, and age subgroups, indicating broad applicability.

Mechanistic biomarker analyses revealed enhanced β-cell function, improved insulin sensitivity, and reductions in fasting glucagon, supporting the dual pancreatic and central mode of action. These results indicate that orforglipron not only lowers glucose but may also preserve or restore β-cell responsiveness, a key determinant of long-term diabetes progression.

### 3.4. Mechanistic Substudies

In-depth mechanistic evaluations employing oral glucose tolerance tests, mixed-meal tolerance tests, and modeling of insulin secretion dynamics showed that orforglipron enhances first-phase insulin secretion, suppresses inappropriate glucagon secretion, and reduces postprandial glycemic excursions [45]. These effects occur in a glucose-dependent manner, minimizing hypoglycemia risk and demonstrating mechanistic fidelity to native GLP-1 physiology.

## 4. Weight Loss and Obesity Outcomes

### 4.1. Phase 2 Evidence

Weight loss is a critical target in T2D and obesity management, given its impact on glycemic control, cardiovascular risk, and metabolic health. Phase 2 trials of orforglipron demonstrated that the compound produces clinically meaningful reductions in body weight in a dose-dependent manner. In adults with T2D, participants receiving the higher dose regimens achieved mean body weight reductions of 7–10% at 26 weeks, with progressive weight loss observed over the study period [29]. The observed weight reduction was associated with improved glycemic parameters, suggesting a synergistic benefit between weight loss and glucose lowering. Importantly, reductions in caloric intake and appetite were noted, indicating central nervous system engagement consistent with the known pharmacology of GLP-1 receptor activation. Gastrointestinal adverse events, including nausea and vomiting, were the primary contributors to early weight loss but were generally mild, transient, and rarely led to discontinuation.

### 4.2. ATTAIN Trials

The ATTAIN program further evaluated the efficacy of orforglipron for weight management in both diabetic and non-diabetic populations. ATTAIN-1 enrolled overweight or obese adults without diabetes, while ATTAIN-2 included participants with T2D. Across both trials, orforglipron produced significant, sustained weight reductions over 72 weeks, with the highest dose yielding mean body weight loss of 12–15% in non-diabetic participants and 10–12% in T2D populations [25,30,46,47]. Notably, a substantial proportion of participants achieved ≥10% weight loss, a threshold associated with meaningful metabolic improvements and cardiovascular risk reduction. Weight reduction was accompanied by favorable changes in waist circumference, body composition, and metabolic biomarkers, reflecting improvement in visceral adiposity and insulin resistance (please see also Section 6.3 below).

These trials also highlighted that weight loss with orforglipron is not solely attributable to gastrointestinal side effects, as progressive reductions were observed after initial dose titration, and appetite suppression was corroborated by patient-reported satiety measures. This observation confirms central engagement of GLP-1 pathways, enhancing energy expenditure and reducing caloric intake, consistent with preclinical mechanistic studies [23,48].

### 4.3. Comparative Context

While injectable GLP-1 RAs have demonstrated similar efficacy, the oral availability of orforglipron provides a substantial advantage in adherence and patient preference. Editorial analyses emphasize that this nonpeptide oral agent challenges the paradigm that oral therapies cannot replicate injectable GLP-1 RA efficacy [39]. Meta-analyses of randomized controlled trials corroborate these findings, demonstrating pooled weight reductions of 9–12% relative to placebo at 26–72 weeks [49,50]. These reductions are clinically meaningful, as a 5–10% weight loss is associated with improvements in HbA1c, blood pressure, lipid profile, and other metabolic parameters.

### 4.4. Safety Considerations

Gastrointestinal adverse events remain the primary limitation for tolerability, with nausea, diarrhea, and vomiting occurring in a dose-dependent manner. However, most events are transient and self-limited, with very few participants requiring dose reduction or discontinuation. Importantly, hypoglycemia remains rare in non-insulin-treated populations, confirming that weight loss and glycemic improvements occur without excessive risk.

Semaglutide and other peptide GLP-1 receptor agonists have been associated, in rare instances, with acute pancreatitis and ocular complications, including non-arteritic anterior ischemic optic neuropathy (NAION) and worsening diabetic retinopathy, as documented in post-marketing surveillance and regulatory safety reviews [51,52,53,54,55,56,57,58,59,60]. These events are thought to reflect either direct GLP-1R–mediated effects or indirect consequences of rapid metabolic and hemodynamic changes (e.g., glycemic shifts, pancreatic enzyme dynamics, or microvascular perfusion), although causality remains incompletely established and absolute risk is very low. 

In contrast, the small-molecule orforglipron has not shown signals of pancreatitis, retinal detachment, or ischemic optic neuropathy. Its observed safety profile has been dominated by dose-dependent gastrointestinal adverse events, consistent with GLP-1R agonism [61,62]. While these data suggest no current evidence of the rare pancreatic or ocular events reported with semaglutide, continued large-scale exposure and post-approval pharmacovigilance will be essential to determine whether such risks represent a class effect or are specific to certain GLP-1RA modalities.

The favorable tolerability profile of orforglipron, combined with oral administration, is likely to enhance real-world adherence and patient satisfaction, a critical factor in long-term obesity management. The main phase 3 trial are reported in Table 3.

### 4.5. Meta-Analytic Evidence

Recent meta-analyses of randomized controlled trials confirm the glycemic efficacy of orforglipron, showing weighted mean reductions in HbA1c of 1.3–1.6% versus placebo, consistent with injectable GLP-1 RAs [61]. These analyses also highlight improvements in fasting glucose, postprandial glucose, and markers of β-cell function, providing robust evidence for efficacy across diverse trial populations. Gastrointestinal events are the most common adverse events, but serious adverse events are infrequent, reinforcing a favorable benefit–risk profile (Table 4).

## 5. Safety, Tolerability, and Cardiovascular Biomarkers

### 5.1. Gastrointestinal and Hypoglycemia Risk

Across Phase 1–3 studies, orforglipron demonstrated a predictable safety profile. Gastrointestinal adverse events, primarily nausea, vomiting, and diarrhea, were dose-dependent and typically mild to moderate in severity [62]. Importantly, these events often resolved with continued treatment or dose titration. Hypoglycemia was uncommon and primarily observed in patients concomitantly receiving insulin secretagogues or insulin, consistent with the glucose-dependent mechanism of action. Severe hypoglycemia was rare, supporting a favorable risk-benefit profile for both diabetic and non-diabetic populations.

### 5.2. Cardiovascular Biomarkers

Beyond glycemic control and weight reduction, orforglipron has demonstrated beneficial effects on cardiovascular (CV) risk biomarkers. In pooled analyses from T2D and obesity trials, treatment was associated with reductions in systolic and diastolic blood pressure, triglycerides, low-density lipoprotein cholesterol, and inflammatory markers, along with increases in high-density lipoprotein cholesterol [62]. These changes were observed as early as 12 weeks and persisted throughout the study duration. Although direct evidence from hard CV outcome trials is not yet available, these biomarker improvements suggest potential cardiometabolic benefit, aligning with the observed effects of injectable GLP-1 RAs on cardiovascular endpoints (Table 5).

### 5.3. Hepatic and Renal Safety

Across the clinical development program, orforglipron has demonstrated a reassuring hepatic and renal safety profile, an important consideration given the high prevalence of chronic kidney disease and nonalcoholic fatty liver disease among individuals with T2D and obesity [30,62,63,64,65,66]. In Phase 1 pharmacokinetic studies conducted in healthy participants, no clinically meaningful elevations in alanine aminotransferase, aspartate aminotransferase, alkaline phosphatase, or total bilirubin were observed following single or multiple ascending doses of orforglipron, indicating an absence of acute hepatocellular or cholestatic toxicity [41,63,67]. These findings were subsequently corroborated in Phase 1b and Phase 2 studies enrolling participants with T2D, in whom baseline hepatic enzyme abnormalities are common due to metabolic liver disease [42,68].

From a mechanistic perspective, the favorable hepatic safety profile is consistent with the pharmacokinetic properties of orforglipron. Detailed disposition analyses demonstrate that the compound undergoes limited hepatic metabolism and does not rely heavily on cytochrome P450–mediated pathways, thereby reducing the risk of hepatotoxic metabolites or clinically relevant drug–drug interactions [41]. Furthermore, exposure–response analyses indicate no relationship between systemic drug exposure and changes in liver enzymes, supporting a wide therapeutic window.

Renal safety has been similarly reassuring. Across Phase 1–3 trials, treatment with orforglipron was not associated with clinically significant changes in serum creatinine, estimated glomerular filtration rate, or urinary albumin excretion. Pharmacokinetic analyses indicate that renal clearance accounts for only a minor fraction of total drug elimination, with the majority of clearance occurring via nonrenal pathways, thereby limiting drug accumulation in the setting of mild to moderate renal impairment [41]. Indeed, orforglipron is eliminated predominantly through hepatic metabolism, with Phase I oxidative biotransformation as the principal clearance mechanism. CYP-mediated metabolism (primarily involving CYP3A) is responsible for most systemic clearance, supporting a terminal half-life compatible with once-daily dosing. Phase II metabolism (e.g., conjugation reactions) occurs downstream of Phase I oxidation but does not appear to be rate-limiting or a major determinant of exposure, functioning mainly to facilitate excretion of metabolites. 

Renal elimination of unchanged orforglipron is minimal, with excretion occurring largely as metabolites via hepatic pathways. This elimination profile suggests predictable pharmacokinetics, limited dependence on renal function, and manageable drug–drug interaction risk consistent with metabolism-driven clearance. These observations align with clinical data from larger efficacy trials, including ACHIEVE-1 and ATTAIN studies, in which renal function remained stable throughout treatment despite substantial weight loss and glycemic improvement [27,45,47,62,69].

Importantly, the absence of clinically relevant hepatic or renal toxicity suggests that no dose adjustment is required in patients with mild to moderate hepatic or renal dysfunction, a practical advantage in routine clinical practice [34]. However, it should be emphasized that patients with severe hepatic impairment or advanced chronic kidney disease were underrepresented or excluded from pivotal trials. Dedicated pharmacokinetic and safety studies in these populations are therefore warranted to define appropriate dosing strategies and fully characterize long-term safety.

### 5.4. Comparative Safety

When evaluated in the context of established injectable GLP-1 RAs, orforglipron exhibits a broadly comparable safety and tolerability profile, with gastrointestinal adverse events constituting the most frequent treatment-emergent effects [70,71,72,73]. Across Phase 2 and Phase 3 trials, nausea, vomiting, and diarrhea occurred in a dose-dependent manner and were most prominent during the initial titration phase, mirroring the class effects observed with peptide-based GLP-1 RAs such as semaglutide and liraglutide [26]. Importantly, these events were generally mild to moderate in severity, transient, and infrequently led to treatment discontinuation.

The risk of hypoglycemia with orforglipron remains low and is largely confined to patients receiving concomitant insulin or insulin secretagogues, consistent with its glucose-dependent mechanism of action. Severe hypoglycemia has been rare across trials, reinforcing the safety of orforglipron in both diabetic and non-diabetic populations [27,39,43,74,75,76]. This safety characteristic is particularly relevant in elderly patients and those with fluctuating glycemic control, in whom hypoglycemia represents a major clinical concern.

Of course, a distinguishing advantage of orforglipron relative to injectable GLP-1 RAs lies in its oral route of administration. By eliminating the need for subcutaneous injection, orforglipron circumvents injection-site reactions, needle-related anxiety, and logistical barriers associated with parenteral therapy. These factors are known to negatively influence adherence and persistence with injectable treatments in real-world settings. Consequently, oral administration is expected to translate into improved long-term adherence and broader acceptance, particularly among patients reluctant to initiate injectable therapy [77,78,79,80]. Another important comparative safety consideration is immunogenicity. Peptide-based GLP-1 RAs can induce anti-drug antibodies, which in some cases may attenuate efficacy or contribute to injection-site reactions. As a nonpeptide small molecule, orforglipron does not elicit an immune response, thereby eliminating the risk of antibody formation and representing a theoretical and practical safety advantage over peptide therapies [81,82,83].

So, available evidence indicates that orforglipron achieves a safety profile that is at least comparable to injectable GLP-1 RAs while offering additional advantages related to route of administration and lack of immunogenicity [47,84,85]. These attributes strengthen its potential role as a first-line or early add-on therapy for T2D and obesity, particularly in patients for whom injectable treatments are undesirable or poorly tolerated.

## 6. Limitations, Knowledge Gaps, and Future Directions

### 6.1. Limitations of Current Evidence

Despite the strength and internal consistency of the available efficacy and safety data, the current evidence base for orforglipron is subject to several important limitations that must be acknowledged when interpreting its clinical potential. Foremost among these is the relatively short duration of most completed trials. The majority of Phase 2 and Phase 3 studies have treatment periods ranging from approximately 26 to 72 weeks, which is sufficient to establish short- to mid-term glycemic and weight effects but inadequate for assessing long-term durability, late-emerging adverse events, or sustained cardiometabolic benefit. This limitation is particularly relevant in the context of chronic diseases such as T2D and obesity, where therapies are typically administered for many years or decades. Experience with other GLP-1 RAs suggests that long-term adherence, tolerability, and efficacy may diverge substantially from results observed in controlled trial settings over shorter time horizons [86,87,88,89,90,91].

Another notable limitation relates to trial population characteristics. Indeed, elderly patients, adolescents, individuals with severe obesity, and those with advanced renal or hepatic impairment have generally been excluded or minimally represented. These exclusions may complicate extrapolation to high-risk populations frequently encountered in real-world practice. Such gaps mirror historical limitations observed in the development programs of injectable GLP-1 RAs and underscore the need for targeted studies addressing vulnerable or understudied groups [78].

Finally, real-world effectiveness data are currently lacking. While randomized trials provide essential evidence of efficacy under idealized conditions, they do not fully capture adherence, persistence, dose interruptions, or discontinuation patterns that ultimately determine population-level benefit. Oral administration is hypothesized to improve long-term adherence compared with injectable therapies, but this assumption remains untested in pragmatic or observational studies. The absence of real-world data therefore represents a critical evidence gap, particularly as clinicians and policymakers consider how orforglipron should be positioned within existing treatment algorithms.

### 6.2. Mechanistic Knowledge Gaps

Although the pharmacological basis of orforglipron has been well characterized in preclinical systems, several mechanistic questions remain unresolved and merit further investigation. One key issue concerns the relative contributions of weight-dependent and weight-independent mechanisms to glycemic improvement. Clinical trial data demonstrate substantial reductions in glycated hemoglobin that correlate with weight loss, but the temporal dissociation observed in some studies suggests that direct β-cell and α-cell effects may account for a meaningful proportion of early glycemic benefit. Quantifying these relative contributions will require carefully designed mechanistic trials incorporating hyperglycemic clamps, mixed-meal tolerance tests, and advanced modeling approaches.

Central mechanisms of appetite regulation also remain incompletely understood. Preclinical studies indicate that nonpeptide allosteric GLP-1 receptor agonists penetrate the central nervous system and engage hypothalamic and brainstem circuits involved in satiety and food reward, but the specific neuronal populations and signaling pathways involved have not been fully delineated. Moreover, it remains unclear whether chronic administration leads to adaptive changes in other central signaling pathways that could influence long-term weight trajectories or treatment responsiveness.

Another important unanswered question relates to β-cell preservation. Peptide GLP-1 RAs have been shown in preclinical models to reduce β-cell apoptosis and enhance β-cell function, but convincing evidence of disease-modifying effects in humans remains limited. Whether orforglipron exerts similar protective effects on β-cell mass or function over prolonged treatment periods has not yet been established. Addressing this question will require long-term studies incorporating biomarkers of β-cell stress, insulin secretory capacity, and possibly imaging-based approaches.

At a more fundamental level, the molecular basis of nonpeptide allosteric GLP-1 receptor activation remains an area of active investigation. While structural studies have provided important insights into binding topology and receptor conformational changes [30,32,33,36,37,38,39], the extent to which different allosteric ligands induce biased signaling or differential receptor trafficking is not fully resolved. A deeper understanding of these mechanisms could inform the rational design of next-generation oral incretin therapies with optimized efficacy, tolerability, or tissue selectivity.

### 6.3. Comparative Effectiveness

A major limitation of the current evidence base is the absence of direct head-to-head comparisons between orforglipron and established injectable GLP-1 RAs or newer dual and triple incretin agonists. Cross-trial comparisons suggest broadly comparable glycemic efficacy and clinically meaningful weight loss, but differences in trial design, patient populations, dosing strategies, and endpoints preclude definitive conclusions (Table 6).

Direct comparative trials would be essential to clarify relative efficacy, tolerability, and treatment persistence, particularly in patients with suboptimal responses to first-line incretin therapy.

Comparative effectiveness studies would also allow more precise evaluation of adherence advantages conferred by oral administration. While oral delivery is intuitively appealing, adherence is influenced by multiple factors, including gastrointestinal tolerability, dosing complexity, and patient expectations. Whether the convenience of oral dosing translates into superior long-term persistence compared with weekly injectables remains an empirical question that can only be answered through head-to-head or real-world comparative studies.

Economic considerations further underscore the need for comparative analyses. Cost-effectiveness evaluations incorporating drug acquisition costs, monitoring requirements, and downstream healthcare utilization will be critical for informing reimbursement decisions and guideline recommendations. As healthcare systems increasingly prioritize value-based care, such analyses will play a decisive role in determining the clinical positioning of orforglipron relative to competing incretin-based therapies.

### 6.4. Cardiovascular and Renal Outcomes

Although early-phase trials of orforglipron have demonstrated favorable effects on cardiovascular risk markers, including reductions in body weight, systolic blood pressure, and atherogenic lipid fractions, these surrogate improvements cannot be assumed to translate into reductions in hard cardiovascular outcomes. Dedicated cardiovascular outcome trials are therefore essential to establish long-term safety and efficacy in patients at high cardiovascular risk, as has been done for several injectable GLP-1 RAs [92,93,94,95,96,97].

Similarly, potential renal benefits remain speculative. While GLP-1 receptor activation has been associated with reductions in albuminuria and slowing of estimated glomerular filtration rate decline in some studies, these effects have not been systematically evaluated for orforglipron in large, long-duration trials. Given the high burden of diabetic kidney disease and the growing emphasis on renoprotective therapies, dedicated renal outcome studies would substantially strengthen the clinical evidence base and clarify the broader impact of oral GLP-1 receptor agonism beyond glycemic control.

### 6.5. Potential for Combination Therapy

Combination therapy represents an important frontier for the clinical application of orforglipron. Given its complementary mechanism of action, orforglipron is well suited for use alongside agents targeting other aspects of metabolic pathophysiology, including SGLT2 inhibitors, basal insulin, or insulin sensitizers [77,79,98]. Early mechanistic and clinical studies suggest additive or synergistic effects on glycemic control and weight loss when GLP-1 receptor agonists are combined with SGLT2 inhibition, without a proportional increase in hypoglycemia risk [85,99,100].

The oral formulation of orforglipron may further facilitate combination strategies by simplifying treatment regimens and reducing therapeutic inertia. In patients with advanced disease or refractory metabolic control, strategic combination therapy could allow individualized targeting of fasting hyperglycemia, postprandial excursions, and excess adiposity. Nonetheless, formal combination trials are needed to define optimal sequencing, dosing, and safety in complex clinical scenarios.

### 6.6. Emerging Formulations and Dosing Strategies

Ongoing research efforts are exploring strategies to further optimize the delivery and tolerability of orforglipron. These include evaluation of extended-release formulations designed to reduce peak-related gastrointestinal adverse events while maintaining therapeutic exposure, as well as alternative dosing schedules that may enhance flexibility and adherence. Pediatric-specific development programs are also under consideration, reflecting the growing recognition of obesity and T2D in younger populations and the unmet need for effective oral therapies in these groups.

Such innovations highlight the adaptability of small-molecule GLP-1 receptor agonism and underscore the broader implications of orforglipron as a platform rather than a single therapeutic entity. Continued refinement of formulation and dosing strategies may further enhance patient-centered care and expand the clinical utility of oral incretin therapies in the years ahead.

## 7. Conclusions

Orforglipron represents a significant advancement in the oral management of T2D and obesity. Its nonpeptide small-molecule design allows once-daily oral administration while achieving glycemic and weight-loss efficacy comparable to injectable GLP-1 RAs. Mechanistic studies confirm enhancement of glucose-dependent insulin secretion, suppression of glucagon, appetite modulation, and central energy regulation. Phase 1–3 trials demonstrate robust HbA1c reductions, meaningful weight loss, favorable cardiovascular biomarker changes, and a predictable safety profile characterized primarily by gastrointestinal events.

The oral formulation may enhance adherence and access, particularly for patients reluctant to use injectable therapies. Nevertheless, knowledge gaps remain regarding long-term efficacy, durability, comparative effectiveness, cardiovascular outcomes, and use in diverse populations. 

Future studies should address these gaps, evaluate combination strategies, and explore extended-release or tailored dosing regimens. Overall, orforglipron is poised to reshape the therapeutic landscape for T2D and obesity, offering a potent, convenient, and well-tolerated alternative to injectable GLP-1 receptor agonists.

## Figures and Tables

**Table 1:** Main Pharmacokinetic Studies of Orforglipron.

Population	Study Design	Dose Range	T_max_ (h)	C_max_ (ng/mL)	Half-Life (h)	Bioavailability (%)	Main Adverse Events	Ref.
**Healthy adults**	Single and multiple ascending dose	1–40 mg	2–3	25–350	24–36	30–35	Mild GI events (nausea, diarrhea)	[28] (Phase 1a)
**T2D**	Multiple-ascending dose, placebo-controlled	10–50 mg	2–4	45–420	26–38	32–40	Mild GI events, transient headache	[29] (Phase 1b)
**Healthy adults**	Oral bioavailability study	20 mg	2	280	28	34	No serious AEs	[41]
**Japanese T2D**	Single and multiple ascending dose	5–50 mg	2–3	35–400	24–36	31–38	Mild GI events, transient nausea	[43]

**Table 2:** Main Phase 2 Dose-Finding Studies, with Glycemic Outcomes.

Population	Doses Tested	Duration	HbA1c Reduction (%)	Fasting Glucose (mg/dL)	Weight Change (%)	Main Adverse Events	Ref.
**T2D, overweight or obese**	2.5, 5, 10, 20, 30, 45 mg once daily	26 weeks	−0.9 to −1.6 (dose-dependent)	−20 to −35	−4.5 to −9.8	Nausea, vomiting, diarrhea; dose-related, mostly mild–moderate	[26]
**T2D**	5, 10, 20, 30, 45 mg once daily	12 weeks	−1.0 to −1.5	−15 to −30	−5 to −8	GI events (nausea, diarrhea), mild headache	[29]
**Early T2D, drug-naïve**	10, 20, 30 mg once daily	40 weeks	−1.3 to −1.5	−25 to −30	−5 to −6	Nausea, diarrhea, transient; mostly mild, early-onset	[27]

**Table 3:** Phase 3 Trials—Glycemic and Weight Efficacy.

Population	Dose	Duration (Weeks)	HbA1c Change (%)	Weight Change (%)	≥10% Weight Loss (%)	Key Adverse Events	Ref.
**Early T2D**	20 mg daily	40	−1.4	−6	35%	Nausea, diarrhea	ACHIEVE-1 [27]
**Obesity, no diabetes**	30 mg daily	26–36	N/A	−12	55%	Nausea, vomiting	ATTAIN-1 [25]
**T2D, obesity**	30 mg daily	72	−1.5	−10	48%	GI events, mild headache	ATTAIN-2 [30]

**Table 4:** Meta-Analyses of Trials Testing Orforglipron.

Population	Number of RCTs/Participants	HbA1c Reduction (%)	Weight Loss (%)	Common Adverse Events	Ref.
**Obese adults ± T2D**	7/2180	−1.3	−9	Nausea, vomiting	[61]
**T2D**	5/1470	−1.4	−7.5	GI events, mild hypoglycemia	[50]
**Obesity**	6/2320	N/A	−10	Nausea, diarrhea	[49]

**Table 5:** Cardiovascular and Metabolic Biomarker Effects.

Population	Duration (Weeks)	Biomarkers	Changes	Clinical Significance	Ref.
**T2D/Obesity**	26–36	SBP, DBP, LDL, HDL, TG, hsCRP	↓ SBP 5–8 mmHg, ↓ LDL 10–15 mg/dL, ↓ TG 15–20%, ↑ HDL 4–6 mg/dL, ↓ hsCRP 15–20%	Suggests improvement in CV risk profile	[25]
**T2D**	40	Fasting insulin, HOMA-IR	↓ Fasting insulin 10–15%, ↓ HOMA-IR 12%	Improved insulin sensitivity and metabolic control	[27]

**Table 6:** Comparative Overview: Orforglipron vs. Injectable GLP-1 RAs.

Drug	Route	HbA1c Reduction (%)	Weight Loss (%)	Key Adverse Events	Dosing
**Orforglipron**	Oral	−1.3 to −1.6	−6 to −12	GI (nausea, vomiting)	Daily
**Semaglutide**	SC	−1.5 to −1.8	−12 to −15	GI, mild pancreatitis	Weekly
**Liraglutide**	SC	−1.0 to −1.5	−6 to −10	GI, injection site reactions	Daily

## Data Availability

No new data were created or analyzed in this study. Data sharing is not applicable. This review was registered in PROSPERO (Prospective Register of Systematic Reviews CRD420261293993).
